# Supplementary material for: Novel Pyridine Bioisostere of Cabozantinib as a Potent c-Met Kinase Inhibitor: Synthesis and Anti-Tumor Activity against Hepatocellular Carcinoma
Source: Int J Mol Sci. 2021 Sep 7;22(18):9685. doi: 10.3390/ijms22189685 (PMC8468607; doi:10.3390/ijms22189685)
Supplement: Supplementary file 1 [file ijms-22-09685-s001.zip › ijms-1345707-supplementary.pdf]

Content

- ^{1}H and ^{13}C NMR spectra
- HPLC chromatograms

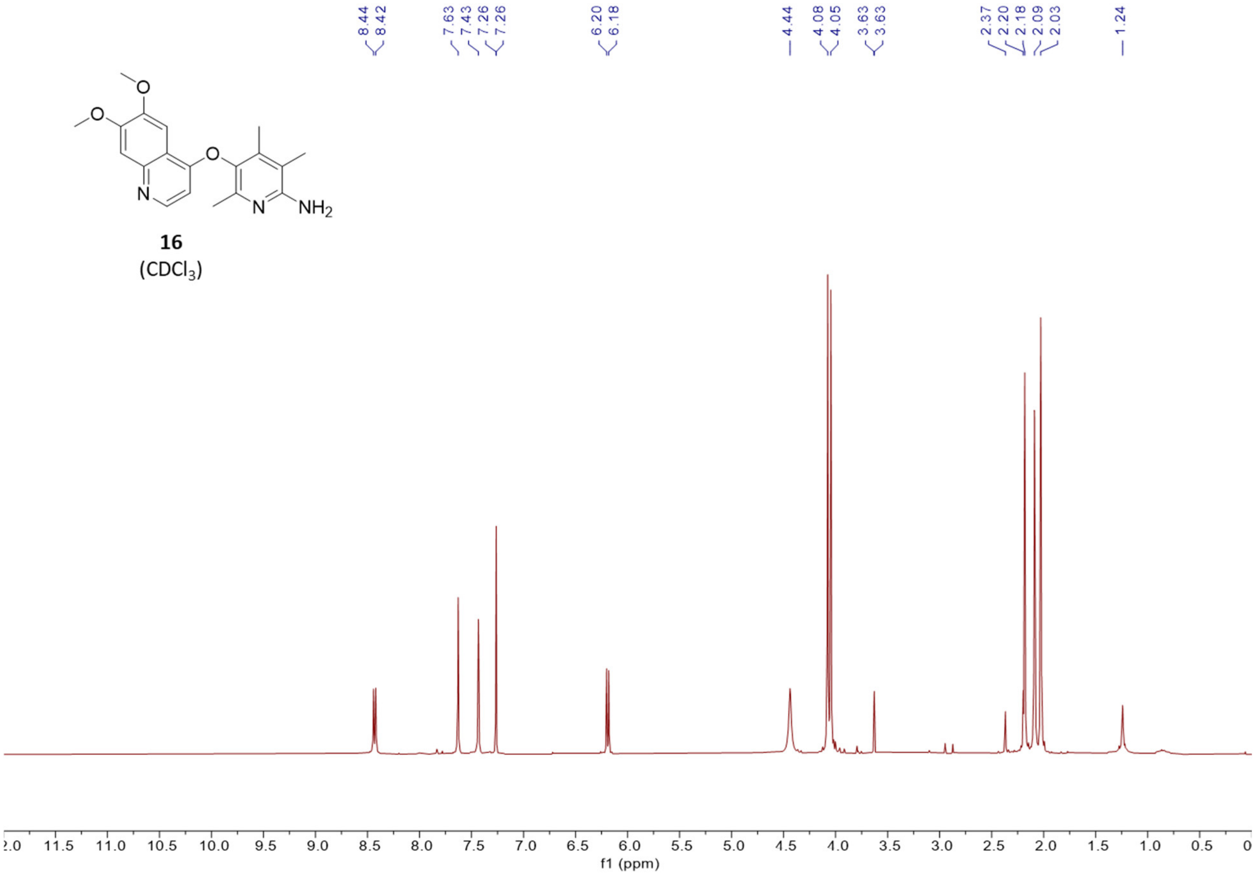

Figure S1. ^{1}H NMR spectrum of compound **16**.

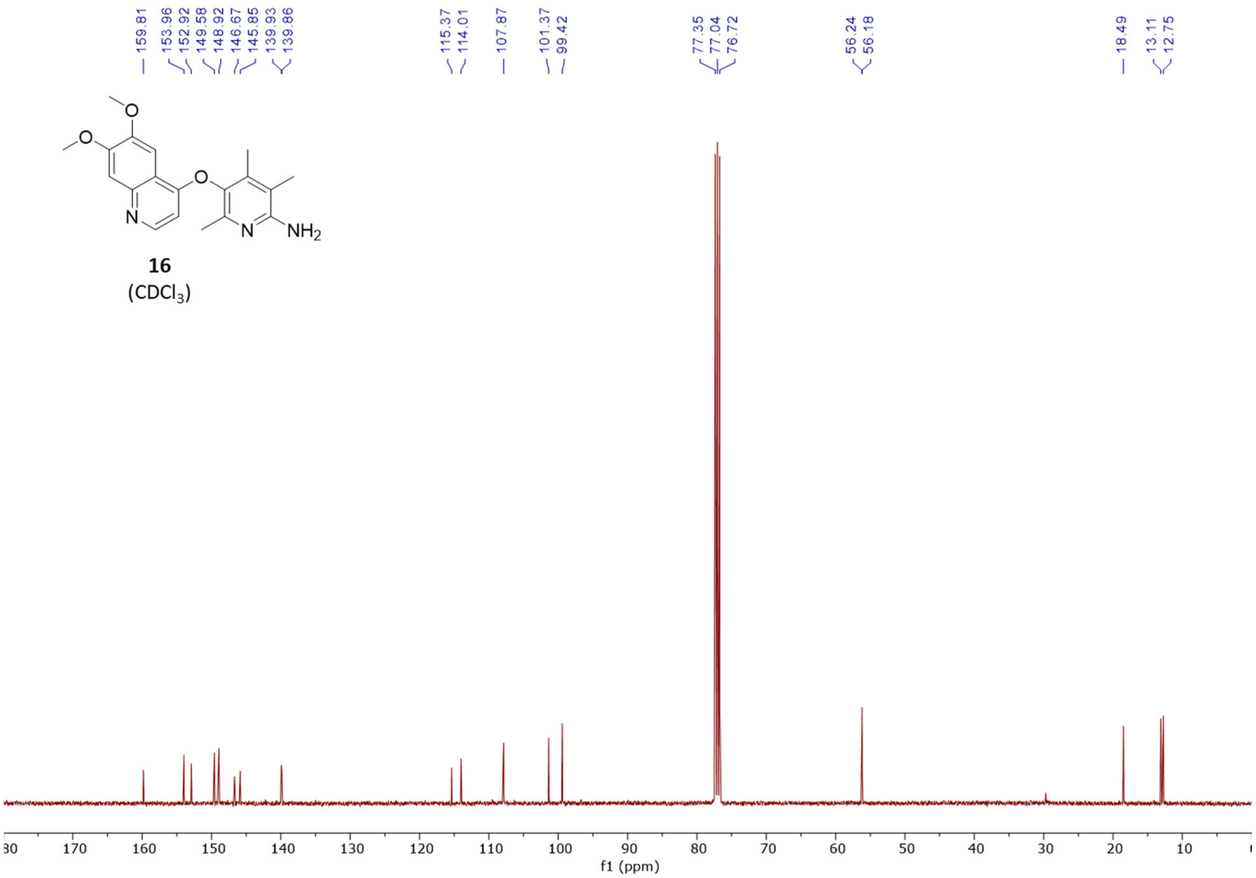

Figure S2. ^{13}C NMR spectrum of compound **16**.

<Chromatogram>

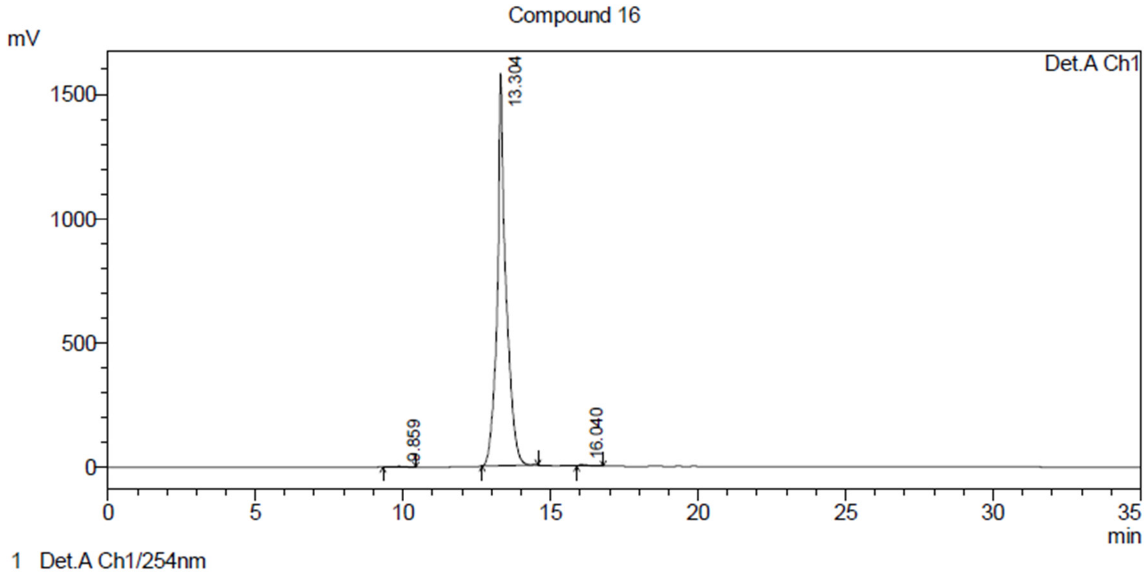

PeakTable

Detector A Ch1 254nm

Peak#	Ret. Time	Area	Height	Area %	Height %
1	9.859	83911	3465	0.255	0.218
2	13.304	32687865	1577560	99.320	99.331
3	16.040	139808	7159	0.425	0.451
Total		32911584	1588184	100.000	100.000

Figure S3. HPLC chromatogram of compound **16**.

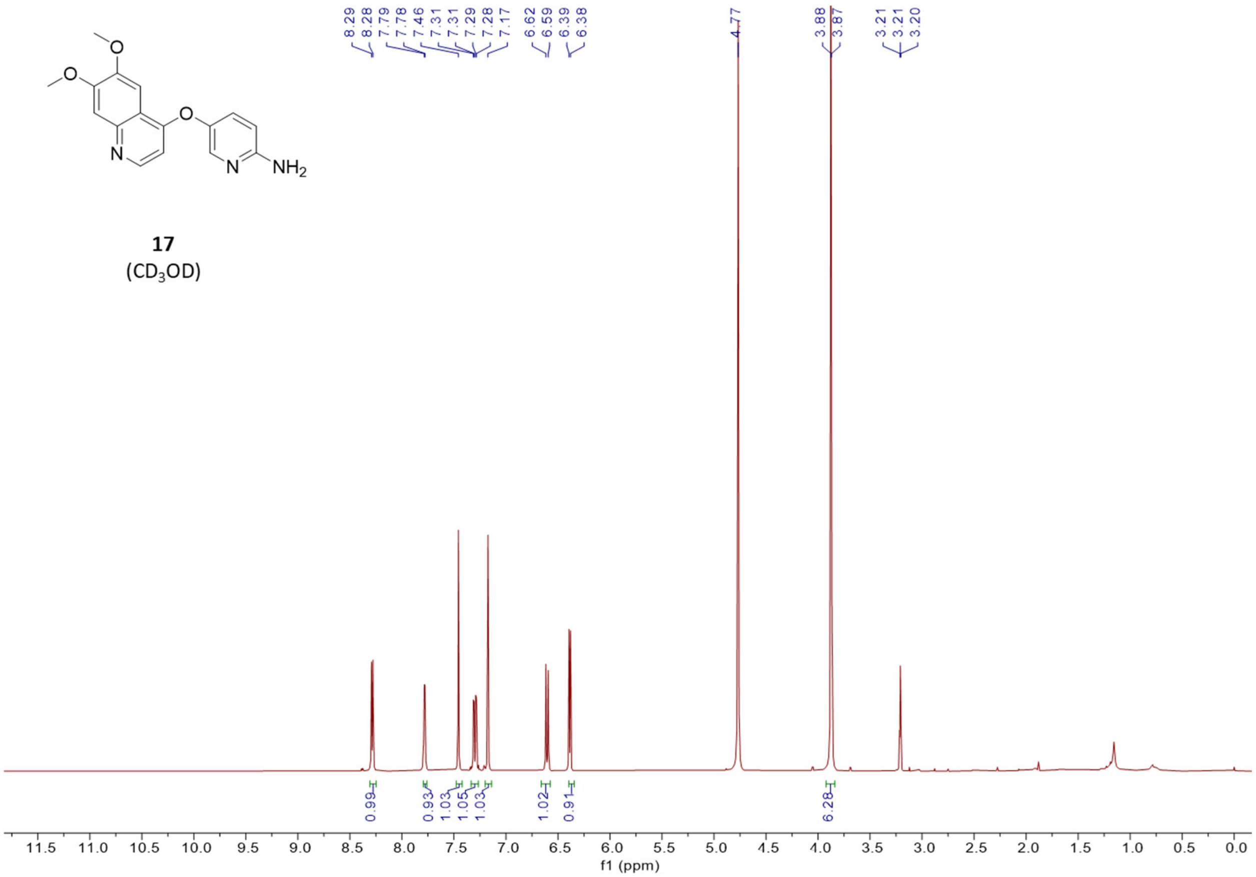

Figure S4. ^{1}H NMR spectrum of compound **17**.

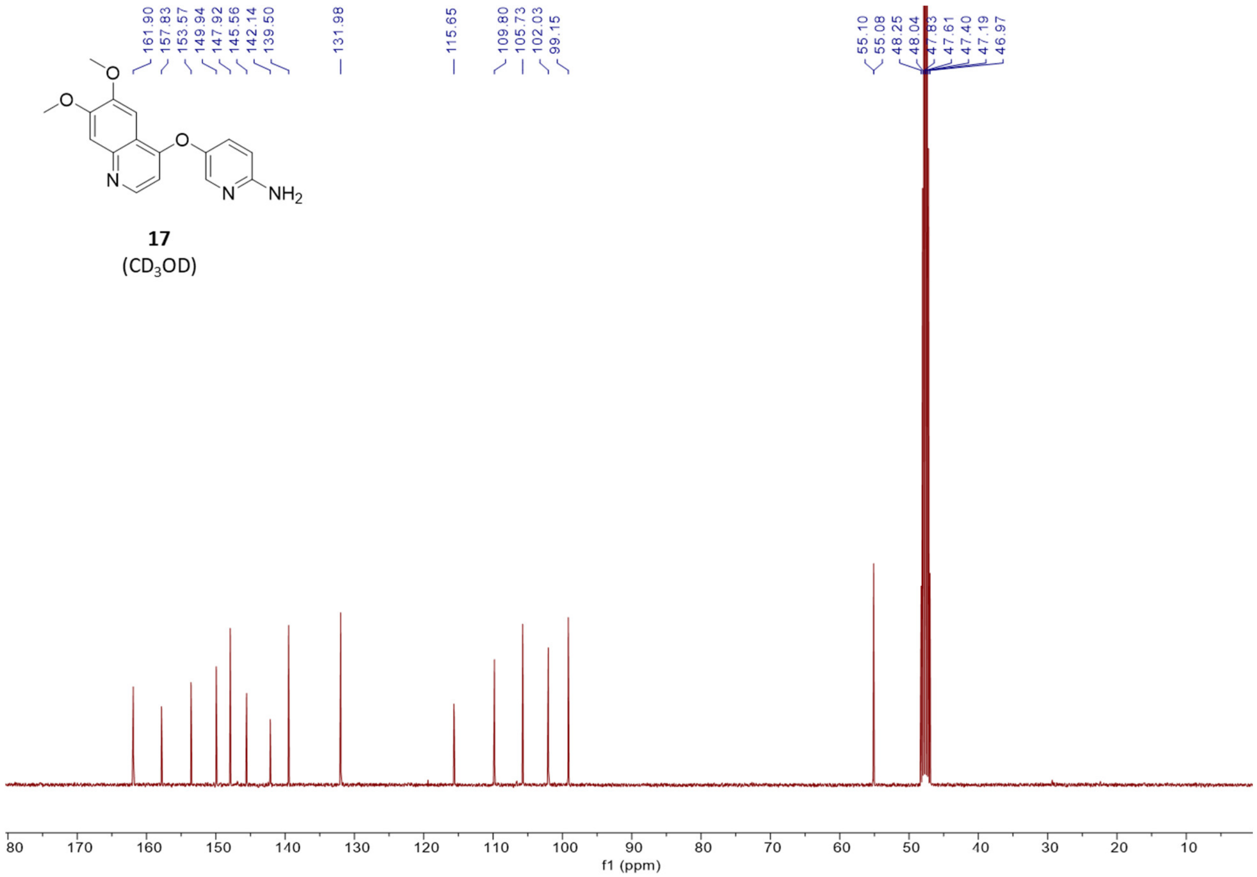

Figure S5. ^{13}C NMR spectrum of compound **17**.

<Chromatogram>

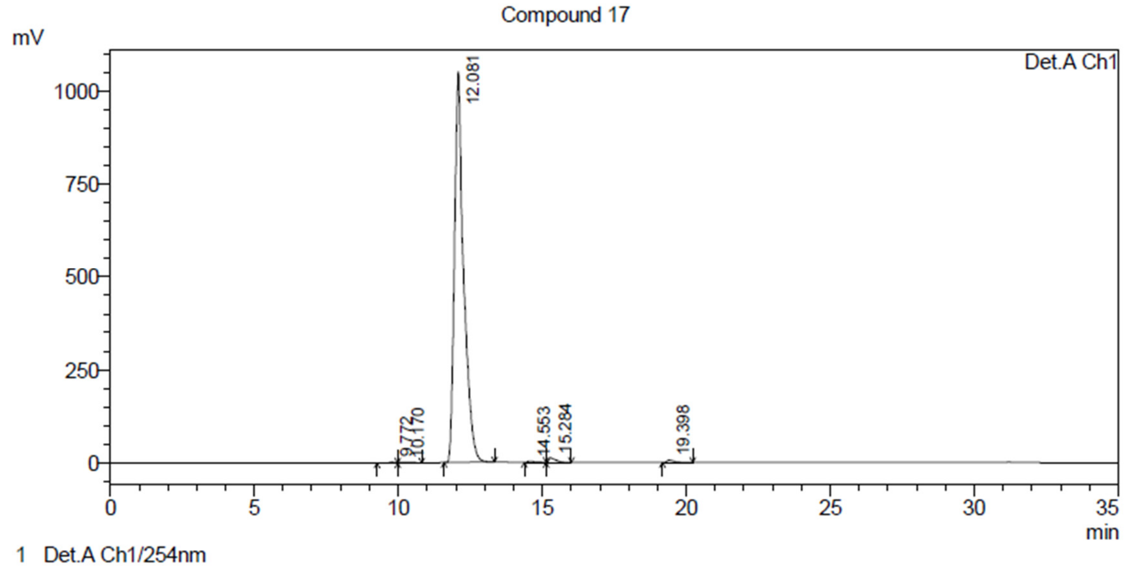

PeakTable

Detector A Ch1 254nm

Peak#	Ret. Time	Area	Height	Area %	Height %
1	9.772	32819	2186	0.138	0.203
2	10.170	43701	1885	0.184	0.175
3	12.081	23258228	1049558	98.128	97.618
4	14.553	61207	3458	0.258	0.322
5	15.284	199670	12324	0.842	1.146
6	19.398	106343	5762	0.449	0.536
Total		23701968	1075173	100.000	100.000

Figure S6. HPLC chromatogram of compound **17**.

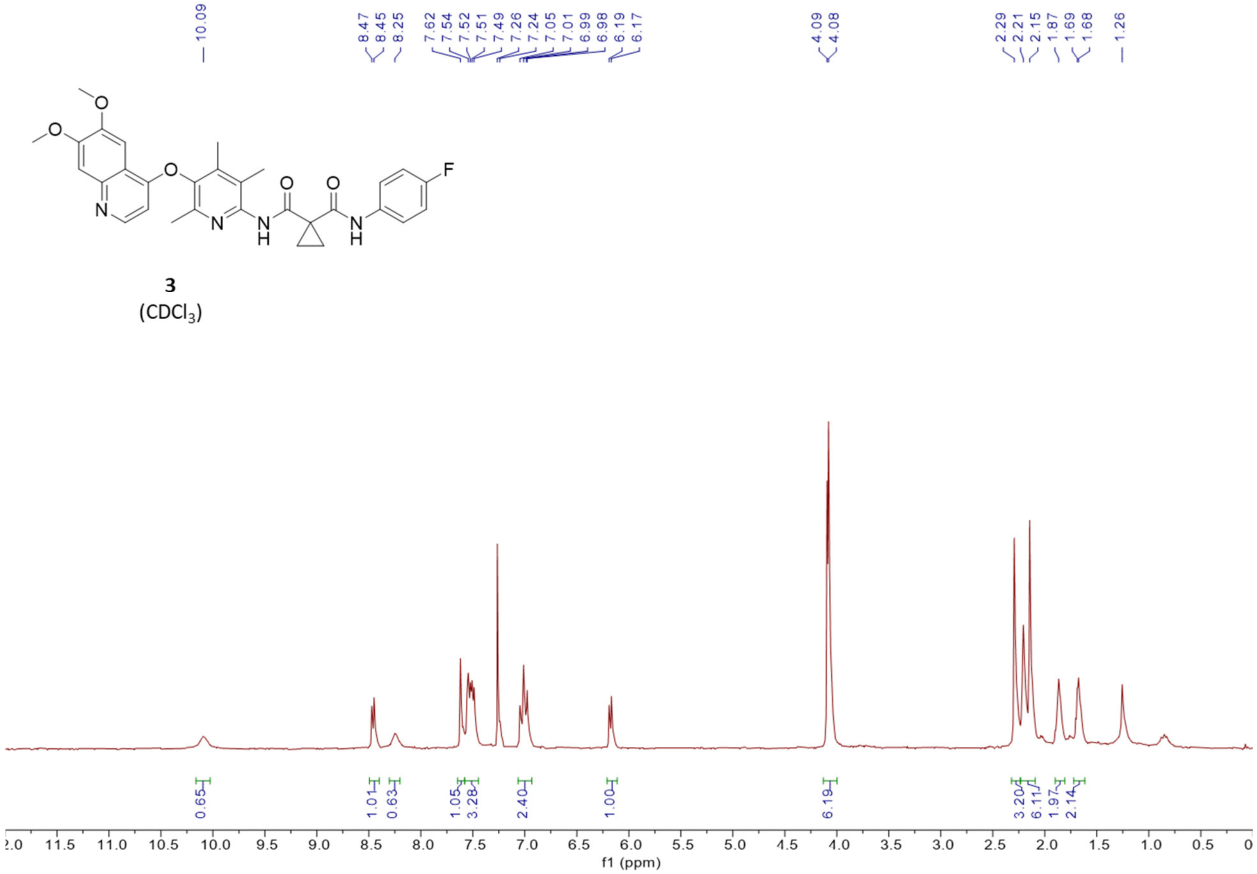

Figure S7. ^{1}H NMR spectrum of compound **3**.

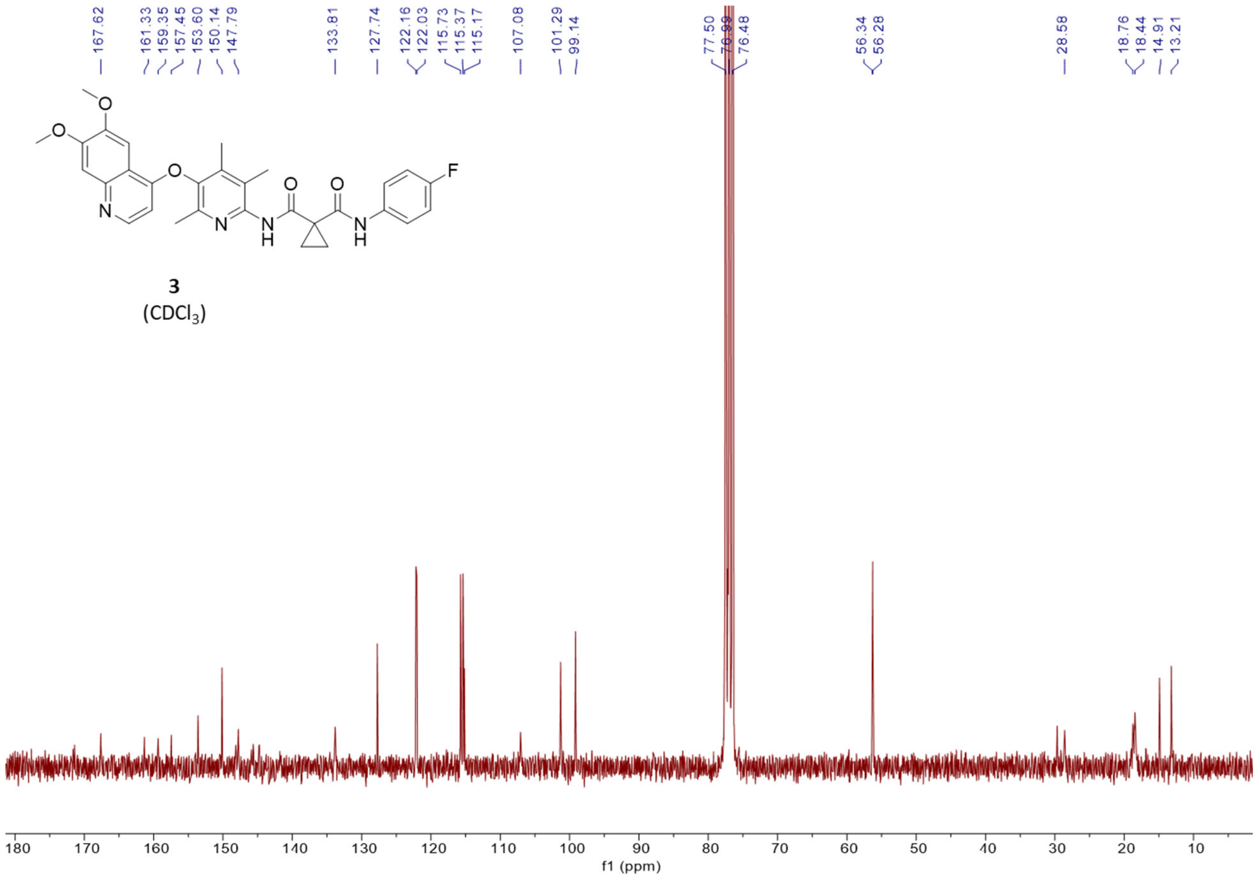

Figure S8. ^{13}C NMR spectrum of compound **3**.

<Chromatogram>

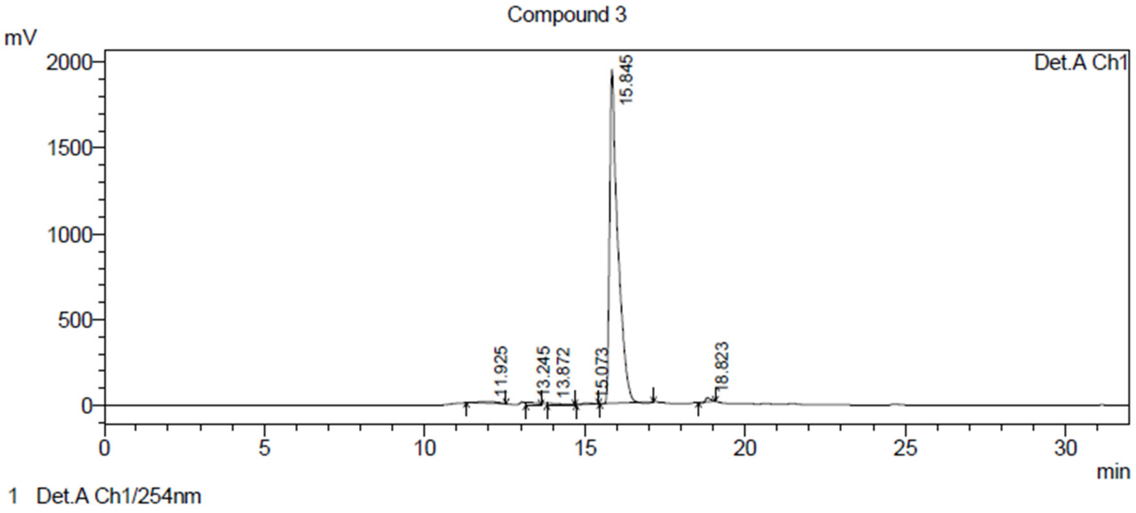

PeakTable

Detector A Ch1 254nm

Peak#	Ret. Time	Area	Height	Area %	Height %
1	11.925	369245	7775	1.025	0.387
2	13.245	288040	13013	0.799	0.647
3	13.872	197914	8927	0.549	0.444
4	15.073	157298	7532	0.437	0.375
5	15.845	34801835	1949231	96.582	96.969
6	18.823	219253	23676	0.608	1.178
Total		36033585	2010154	100.000	100.000

Figure S9. HPLC chromatogram of compound **3**

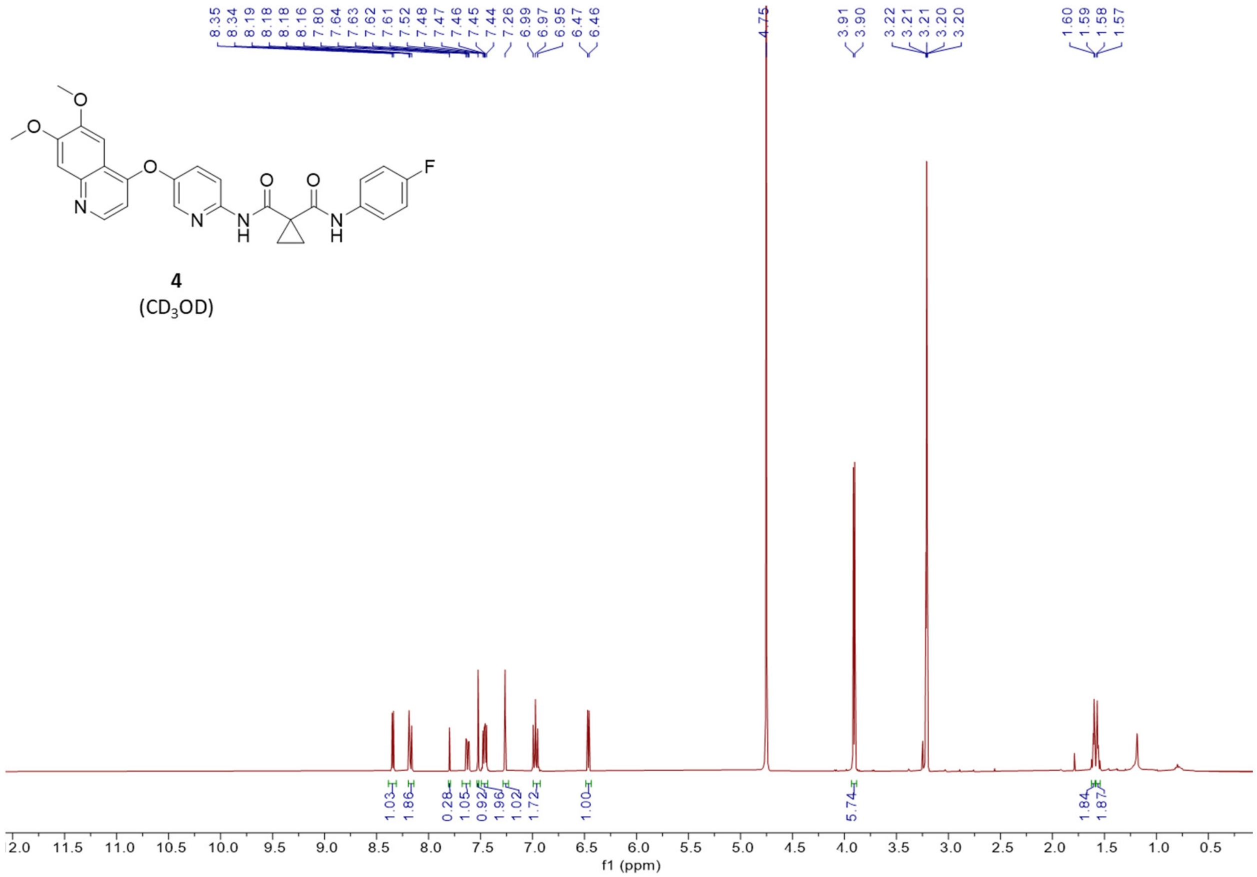

Figure S10. ^{1}H NMR spectrum of compound **4**.

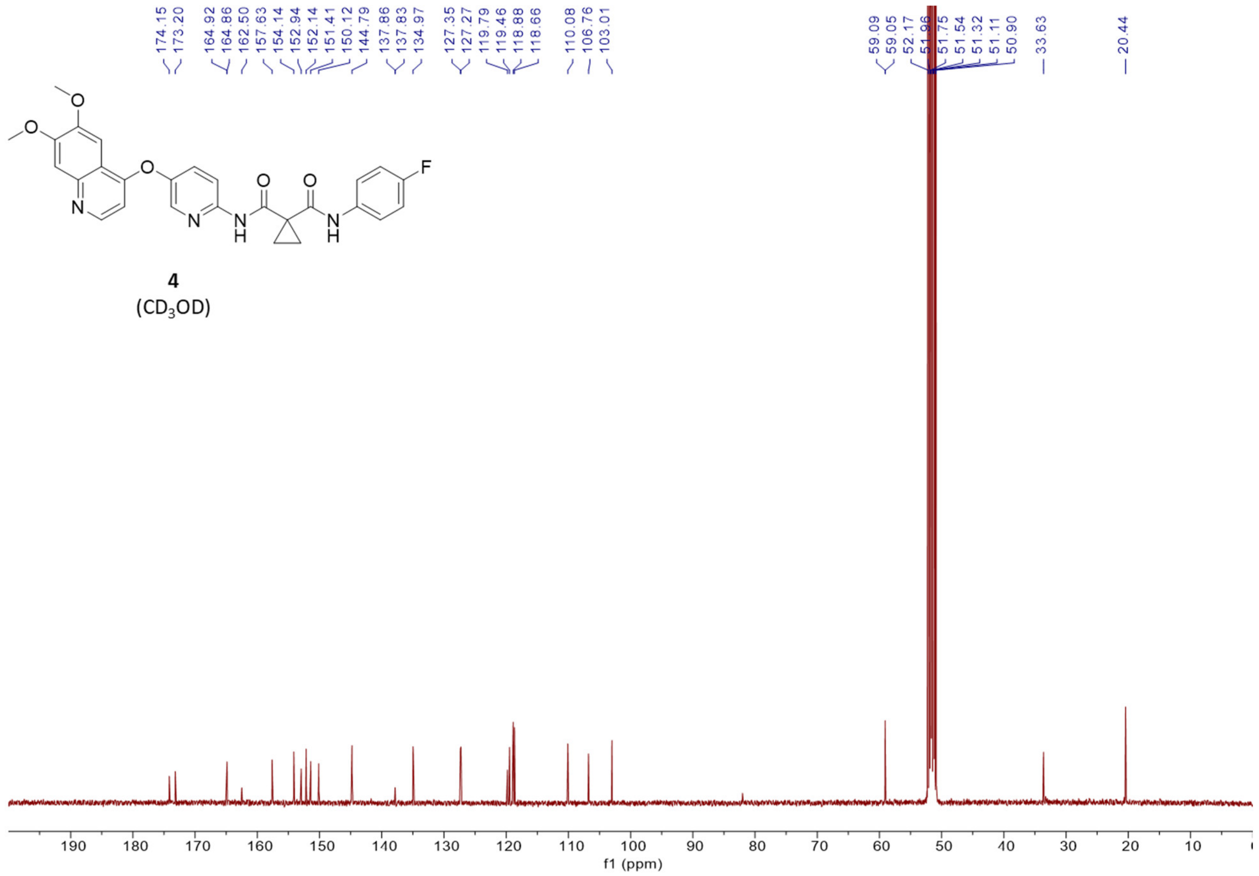

Figure S11. ^{13}C NMR spectrum of compound **4**.

<Chromatogram>

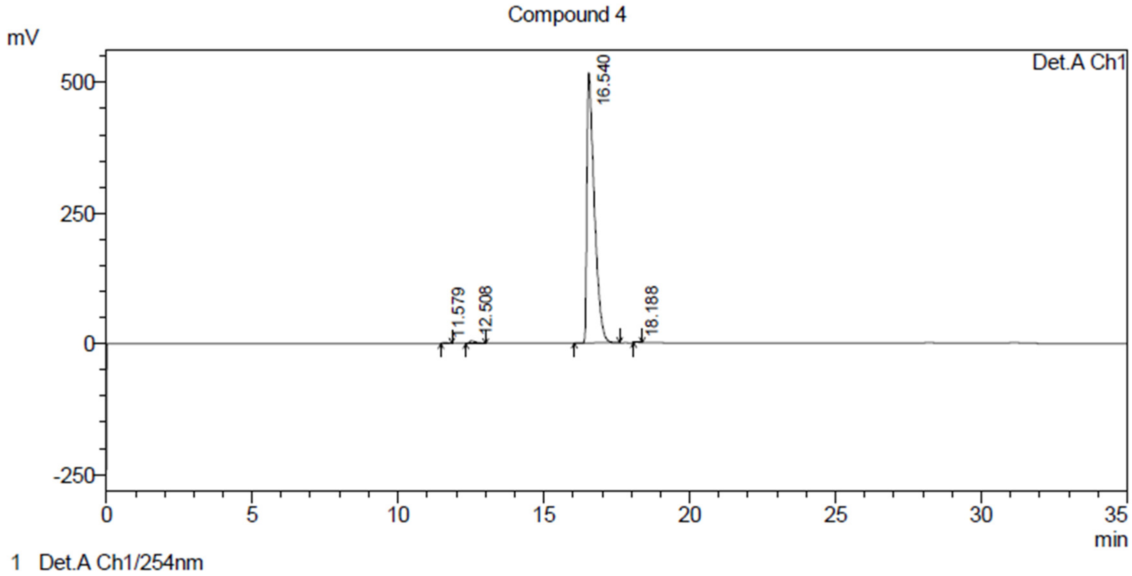

PeakTable

Detector A Ch1 254nm

Peak#	Ret. Time	Area	Height	Area %	Height %
1	11.579	18434	1999	0.199	0.381
2	12.508	83079	4623	0.898	0.881
3	16.540	9138912	517542	98.815	98.599
4	18.188	8111	730	0.088	0.139
Total		9248535	524895	100.000	100.000

Figure S12. HPLC chromatogram of compound **4**.
